# Supplementary material for: Lipoprotein(a) levels in a sample of 115,197 subjects from the largest Brazilian private laboratory
Source: PLoS One. 2025 Jun 27;20(6):e0326365. doi: 10.1371/journal.pone.0326365 (PMC12204575; doi:10.1371/journal.pone.0326365)
Supplement: Attachment — Submitted filename: Response to Reviewers.docx [file pone.0326365.s003.docx]

Dear reviewers,

Thank you for dedicating your time and expertise to provide valuable feedback on our manuscript. We greatly appreciate your insightful suggestions, which have helped us improve the quality of our work. Below, we have provided detailed responses to each of your comments and indicated where revisions were made in the manuscript. Your feedback has been invaluable, and we have strived to address all points comprehensively.

**Journal Requirements:**

**1. Please provide an amended statement that declares *all* the funding or sources of support (whether external or internal to your organization) received during this study. Please also include the statement “There was no additional external funding received for this study.” in your updated Funding Statement. Please also include your amended Funding Statement within your cover letter.**

Thank you for reminding us about the necessary details in the funding description. We have provided the requested detailed information in our cover letter, as well as in the submission system as follows: “Novartis Biociências SA provided financial support for the conduct of the research, preparation of the article, study design, critical analysis, interpretation of data, writing of the report, and the decision to submit the article for publication. There was no additional external funding received for this study.”

**2. Please provide an amended Funding Statement and Competing Interests Statement, including details about the authors' commercial affiliations, their roles in the study, and any restrictions on sharing data and materials. Confirm that the commercial affiliation does not affect adherence to PLOS ONE’s policies on data sharing.** “

We have carefully reviewed and updated these sections in accordance with your guidance as follow: “The authors have disclosed the following potential conflicts of interest: PR is an employee and stockholder of Novartis Biociências SA. PR also holds a leadership role as an Emergent Leader in the World Heart Federation. EGL reports that Novartis made payments to DASA, their current employer, in relation to this manuscript. EGL also received honoraria as a speaker for Novartis in educational events and has served on an advisory board for Bayer. FPPLL received direct payments from Bayer for attending meetings or travel. EF is a salaried employee of Novartis Biociências SA. IC received consulting fees through their institution, ConstatBio, related to DASA. MHCGC, PCG, and AAS declared no competing interests. These commercial affiliations do not alter our adherence to PLOS ONE's policies on data and material sharing.”

**Reviewers’ comments**

**Reviewer 1:**

**1. The study design needs to be standardized. In the Introduction section, it is mentioned that the study design is a retrospective observational study. However, in methodology, the author states that it is cross-sectional, nonintervention observational study.**

Thank you for highlighting this discrepancy in the study design description. We have corrected the manuscript to consistently describe the study design as a retrospective observational study in both the Introduction (page 1, paragraph 1) and Methods sections (page 1, paragraph 2).

**2. What was the Metabolic Syndrome criteria did the author adopt to identify subjects with MetS? This was not described in the methodology which is worthwhile incorporating to give more clarity to the method.**

Thank you for your comment regarding the clarification of the MetS criteria used in our study. The criteria is described on page 4, lines 77-80: *“Based on US National Cholesterol Education Program (NCEP) definitions [10], the following metabolic syndrome laboratorial criteria were considered: triglycerides > 150 mg/dL; fasting plasma glucose > 110 mg/dL; HDL-cholesterol < 40 mg/dL (men), HDL- cholesterol < 50 mg/dL (women).”*

At page 20 and 21 lines 341- 344, we clarify in the discussion that we used only laboratory criteria to Metabolic Syndrome definition -. *“Furthermore, this is the main reason we could not estimate the prevalence of metabolic syndrome and relied exclusively on laboratory-base criteria of metabolic syndrome. Lastly, a limitation of this study is the lack of data on ethnicity, which could impact the distribution of Lp(a) in the sample.”*

**3. Based on the description of determining LDL-c, there are 3 methods which is direct measurement (utilizing 6 equipment), LDL-c calculations by Friedewald and Martin-Hopkins formulas. How do you ensure correlation of results between the 6 equipment? How do you determine the agreement between the direct measurement and calculated LDLc? Are the 2 equations well correlated?**

Thank you for your valuable questions regarding the methods used to determine LDL-c levels and for your thoughtful inquiry into the correlation and agreement between the different techniques and formulas. The decision to include LDL data measured by different formulas and techniques in our study stems from two important premises. Premise 1 highlights that this study was conducted in a real-world setting, utilizing the database of the largest clinical laboratory in Latin America. Unlike a randomized clinical trial, our objective was to capture data from different laboratories and techniques, reflecting the diversity of test methods commonly used in practice.

Premise 2 is based on existing literature, which presents publications from different countries comparing LDL levels calculated by modified Martin/Hopkins estimation or the Friedewald formula with direct homogeneous assay measured low-density lipoprotein cholesterol. It has been observed that the Friedewald equation tends to underestimate LDL-C levels in very high and high-risk settings. An analysis conducted on Hungarian patients concluded that LDL-C estimation using the Martin/Hopkins formula, which is validated by the beta-quantification method, yields a more accurate LDL-C value than that calculated by the Friedewald formula.

By incorporating the data obtained through various measurement methods, particularly with consideration of the limitations of the Friedewald formula, our study aims to provide a comprehensive analysis that reflects real-world scenarios and contributes to the understanding of LDL-C estimation. By doing so, we can gain insights into the accuracy and reliability of different measurement techniques and highlight the importance of using validated formulas in specific clinical contexts.

It is important to acknowledge that the inclusion of data from different formulas and techniques may introduce variability. However, through appropriate statistical analyses and quality control measures, we can account for these factors and provide valuable information on the correlation, agreement, and potential biases associated with different measurement methods. Ultimately, this approach strengthens the validity and applicability of our study findings, offering a more robust understanding of LDL-C estimation in real-world settings. A paragraph was added to the article in the discussion section. It is presented on page 19, lines 311–319: "*The decision to include LDL- c and HDL-c data from various formulas and techniques in our study is based on two important premises. Firstly, we aim to conduct the study in a real-world setting, with data obtained from different laboratories and techniques commonly used in practice. Secondly, existing literature supports the need to compare different methods of LDL-c estimation, as the Friedewald formula has been shown to underestimate LDL-c levels in certain scenarios [25,26]. By incorporating diverse measurement methods and addressing potential biases, our study will provide a comprehensive analysis that enhances the understanding of LDL-c and HDL-c estimation in real-world settings."*

**4. Similarly with HDL-c measurement between the equipment. How do you determine the instruments correlated with one another?**

Thank you for your follow-up question regarding the measurement of HDL-c and the correlation between the equipment used. As we explained in our response to Question 3, the approach to ensuring correlation and agreement between the different instruments and measurement techniques applies similarly to HDL-c as it does to LDL-c. The decision to include data from different techniques, based on real-world data from the largest clinical laboratory in Latin America, reflects the diversity of test methods commonly used in clinical practice. This strategy allows for a more comprehensive analysis of the methods' accuracy and reliability. We hope this explanation helps clarify the methodology and thank you again for your attention to detail.

**5. How did the author minimized any bias due to ethnic differences in the Brazilian population, considering that Brazil has approximately 5-7 major ethnic groups from literatures? Worthwhile exploring this or highlighting how any potential bias was overcome.**

Thank you for your question regarding bias due to ethnic. This aspect has been clarified in the text on page 20 and 21, lines 343-362. Here are the key points that address this concern:

The study utilized data from a national database of a private Brazilian laboratory, which included a wide range of ages and geographic regions. This approach ensured a representative sample of the Brazilian population, known for its ethnic diversity. The geographic analysis of patient locations revealed that individuals were mainly concentrated in urban centers such as São Paulo, Rio de Janeiro, Brasília, Curitiba, Recife, and Fortaleza. This suggests that the sample included individuals from diverse ethnic backgrounds, reflecting Brazil's demographic composition.

Although the study did not collect specific data on ethnicity, it acknowledges Brazil's ethnic heterogeneity and the possible influence of this diversity on Lp(a) levels. International studies indicate that Lp(a) levels tend to be higher in non-white populations, which includes a significant portion of the Brazilian population. The document references previous studies that investigated the prevalence of genetic polymorphisms associated with Lp(a) in different ethnic groups in Brazil. These studies showed that ethnicity in Brazil is highly heterogeneous, including individuals of European, African, and Amerindian ancestry.

The study also acknowledges its limitations, including the lack of data on ethnicity, and suggests that future research should explore the influence of ethnic diversity on Lp(a) levels in the Brazilian population in more detail. This includes collaborations with medical societies and medical education programs to establish appropriate guidelines for requesting and managing Lp(a) tests.

These points demonstrate that the author took measures to minimize bias due to ethnic differences by ensuring a representative sample and recognizing the need for future research to address this issue in more detail.

**6. The sentences in lines 271-275 are unclear in terms of what the message the author aims to deliver. Please clarify.**

We appreciate your feedback and have revised the text to ensure the message is clear and concise. The updated version is as follows: *“According to the latest EAS and ACC guidelines [3,4], it is recommended to use Lp(a) as a tool to refine cardiovascular risk, especially when levels exceed 50 mg/dL. Additionally, Lp(a) can help identify individuals with an extremely high risk, similar to Heterozygous Familial Hypercholesterolemia, when levels go beyond 180 mg/dL.”* (Page 17, lines 467 – 470).

**7. Sentence in lines 286-287: 18% of study population refers to what lipid patterns?**

Thank you for your observation. We clarify the information on page 10, lines 180 – 184: *“Based on our dataset, we found that 18% of the individuals (20,859 subjects) had Lp(a) levels above 50 mg/dL, which is considered a high risk for cardiovascular disease. This indicates that Lp(a) assessment alone could classify at least 18% of our sample as having a high cardiovascular risk, regardless of other laboratory exams and comorbidities.”*

**8. What are the future research that can be done to close the gaps of the limitations you've highlighted in the discussion?**

Thank you for your thoughtful question. We have added future research recommendations on page 21, lines 355 - 362: *“In order to address the limitations highlighted in our study, there are several future research avenues that can be explored. In terms of Lp(a) request according to patient profile, collaborations with medical societies and medical education programs could help address these gaps and establish guidelines for the appropriate request and management of Lp(a) testing. Ongoing research in a Brazilian tertiary cardiovascular specialized hospital could also provide valuable insights into Lp(a) in different cardiovascular risk patients and the current treatments for LDL-C.”*

**9. To include quality controls and whether method verification was done prior to the analyses of the biomarkers since the results are heavily lab based and therefore ensuring the reliability of data is important.**

Thank you for your comment. Here are the relevant points that address this concern:

The study utilized data from a national database of a private Brazilian laboratory, Diagnósticos da América S.A. (DASA), which ensured a comprehensive and standardized approach to laboratory measurements. The following quality controls and method verifications were implemented:

1. Laboratorial Measurements: The biomarkers, including Lp(a), fasting blood glucose, A1c, total plasma cholesterol, HDL cholesterol, LDL cholesterol, and triglycerides, were measured using standardized methods and equipment. For instance, Lp(a) was measured in mg/dL by nephelometric assay using Cobas 8000, Imuno BNII Cluster 1, Cluster 2, and Main Cluster devices
2. Reference Intervals and Methods: Each biomarker was measured using specific methods with established reference intervals. For example, fasting plasma glucose was evaluated by the hexokinase method, and A1c was measured using various devices such as Advia Cluster, Cobas, Premier Hb9210, Tosoh Cluster, Cluster GHLV, Hedmato D-100, and Cobas C513. These methods ensured consistency and accuracy in the measurements.
3. Quality Control Procedures: The study adhered to rigorous quality control procedures to ensure the reliability of the data. This included the use of multiple devices and methods to cross-verify the results. For instance, triglycerides were measured using an enzymatic colorimetric method with cholesterol esterase oxidase, and LDL-C was calculated using both the Friedewald formula and the Martin-Hopkins formula
4. Statistical Analysis: The study employed robust statistical methods to analyze the data, including the use of the Kolmogorov-Smirnov test, Mann-Whitney test with Bonferroni correction, Kruskal-Wallis test, and Monte Carlo correction with 1,000 resampling
5. Ethical Considerations: The study followed ethical principles and obtained a waiver of informed consent due to the retrospective nature of the study. All data was anonymized and encrypted to protect patient privacy.

These points demonstrate that the study included comprehensive quality controls and method verifications to ensure the reliability of the data, given the lab-based nature of the results.

**10. Grammatical errors which is recommended to have the manuscript undergo grammar check, particularly in the discussion section.**

Thank you for your feedback regarding the language used in the manuscript. We have reviewed and improved the manuscript.

**11.** **Lp(a) and lipoprotein (a) terms are used interchangeably. Suggest to use Lp(a) throughout once the abbreviation is defined in the introduction.**

Thank you for your valuable suggestion regarding consistent terminology. We have ensured that the term "Lp(a)" is used consistently throughout the manuscript after its definition in the introduction.

**Reviewer 2:**

**1. Expand discussion on limitations;**

Thank you for your suggestion to expand the discussion on limitations. We have addressed this by elaborating on a dedicated section called “Limitations”, where we provide a more in-depth analysis of the study’s constraints and their potential implications.

**2. Compare with different ethnicity among Brazilians;**

Thank you for your suggestion to include a comparison with different ethnicities among Brazilians. We have incorporated this aspect into the discussion as a limitation, and the updated section is as follows: *“Lastly, a limitation of this study is the lack of data on ethnicity, which could impact the distribution of Lp(a) in the sample. While there is limited information available on Lp(a) in the Brazilian population, few data from international registries and clinical trial sub analysis, showed that mean Lp(a) levels tend to be higher in South America, including Brazil, due to the diversity of ethnicity. Non-white patients often have more elevated Lp(a) levels. However, it is worth noting that Lp(a) levels were found to be higher in countries with a larger population of black patients, such as the USA, South Africa, and Brazil. Additionally, a Brazilian study investigating the prevalence of the rs10455872 Lp(a) polymorphism as a predictor for prevalent cardiovascular disease showed that ethnicity in Brazil is highly heterogeneous, comprising various ethnic groups, including individuals of European descent, African descent, and Amerindians. While stratified analysis by race did not support a higher prevalence of rs10455872 polymorphism in a specific ethnic group.”* (page 20, lines 343 - 355).

**3. Any potential clinical implications;**

Thank you for your thoughtful question regarding the potential clinical implications of our findings. We have addressed this point and included the following in the revised manuscript in page 20, lines 321-332: *“The findings of this paper regarding Lp(a) in the Brazilian population have important clinical implications. Lp(a) is known to be a significant risk factor for atherosclerotic cardiovascular disease (ASCVD) and calcific aortic valve stenosis. Currently, there are challenges in the screening and management of Lp(a) in both primary and secondary prevention. However, this study highlights that in Brazil, the majority of Lp(a) testing is being done for primary prevention. Interestingly, only 18% of the tested population presented with high Lp(a) levels, above 50 mg/dL, which is a lower percentage compared to what is seen in the literature for other countries. For this specific group of patients, it is crucial to consider more aggressive monitoring of cardiovascular risk and intensification of the management of overall risk factors. The results of this study may contribute to future clinical practice recommendations and emphasize the importance of proactive management in individuals with high Lp(a) levels in the Brazilian population.”*

**4. Add statement on future research Direction;**

Thank you for your suggestion. We approached future research directions in page 21, lines 355 - 362: *“In order to address the limitations highlighted in our study, there are several future research avenues that can be explored. In terms of Lp(a) request according to patient profile, collaborations with medical societies and medical education programs could help address these gaps and establish guidelines for the appropriate request and management of Lp(a) testing. Ongoing research in a Brazilian tertiary cardiovascular specialized hospital could also provide valuable insights into Lp(a) in different cardiovascular risk patients and the current treatments for LDL-C.”*

**5. Language is a bit lengthy. Perhaps can be more direct and concise. Some parts are repetitive (example line 28-31, 32-35, 67-71, spelling error line 96. Confidence interval can be abbreviated to CI.**

Thank you for your feedback regarding the language and structure of the document. We have reviewed it and made sure all the points you mentioned have been addressed.

**6. Table 2 is difficult to read and understand. Table 3 Suggest adjust column width so that percentages can be one line.**

Thank you for your suggestion regarding Table 3. We have adjusted the column widths.
