# Supplementary material for: Lipoprotein(a) levels in a sample of 115,197 subjects from the largest Brazilian private laboratory
Source: PLoS One. 2025 Jun 27;20(6):e0326365. doi: 10.1371/journal.pone.0326365 (PMC12204575; doi:10.1371/journal.pone.0326365)
Supplement: Attachment — Submitted filename: Response to Reviewers 2_PR.docx [file pone.0326365.s004.docx]

Dear Reviewer,

We sincerely thank you for your careful and constructive evaluation of our manuscript. Your insightful comments were extremely helpful in improving the clarity, precision, and overall quality of our work. Below, we provide a detailed point-by-point response, along with a description of the corresponding changes made to the manuscript.

**Review Comments to the Author**

**Reviewer #3**

1. **Abstract: please include the p-value for the comparison between men and women.**

Thank you for your observation regarding the Abstract. As requested, we have added the p-value for the comparison between men and women to improve clarity and completeness of the statistical reporting.

2. **Introduction, line 59: The phrase “secondary cause of Lp(a) levels alterations” needs clarification. Could the authors specify which secondary causes are being referred to?**

Thank you for pointing out the ambiguity in our phrase “secondary cause of Lp(a) levels alterations.” We appreciate your diligent attention to detail.

The secondary causes we are referring to in this context include certain health conditions and treatments that can affect Lp(a) levels. For instance, physical activity and exercise can influence Lp(a) levels, though the extent varies low values depending on factors such as age, type, intensity, and duration of the exercise.

Hormone replacement therapy (HRT) can reduce Lp(a) levels in postmenopausal women, with oral administration being more effective than transdermal estradiol. The Lp(a)-lowering effect of HRT is not influenced by the type of HRT, the dose of estrogen, or the addition of progestogen.

Kidney diseases can cause increases in Lp(a) levels, depending on disease stages, dialysis methods, and apolipoprotein(a) phenotypes. Conversely, liver diseases may lower Lp(a) levels in tandem with disease progression, although there have been conflicting results about the association between Lp(a) levels and nonalcoholic fatty liver disease in population studies. We modified in the text this sentence.

3. **Lines 87, 88, 90, 108: The unit mg/dL is missing in several instances. Additionally, A1c should be referred to as HbA1c, and it is not expressed in mg/dL. Please remove mg/dL from line 88 and correct the terminology in line 90 accordingly.**

Thank you for your attentive review and valid suggestions. We have taken your comments into consideration and made the necessary amendments in the text. Instances that were missing the unit mg/dL have been corrected and the inaccurate use of A1c is now appropriately referred to as HbA1c. The term mg/dL has been duly removed from line 88.

4. **The US NCEP/ATP III cut-off values are inaccurately reported. Triglycerides should be defined as ≥150 mg/dL, and fasting glucose as >110 mg/dL. Please revise these values.**

Thank you for your keen observation regarding the inaccuracies in the reported cut-off values for triglycerides and fasting glucose according to the US NCEP/ATP III. We have amended these values in our text, with triglycerides now correctly defined as ≥150 mg/dL and fasting glucose accurately stated as ≥110 mg/dL. Your precise attention to detail helps maintain the integrity and accuracy of our study.

5. **HbA1c: Ensure that "A1c" is consistently reported as HbA1c throughout the manuscript.**

Thank you for your attentive review and valid suggestions. We have been corrected and the inaccurate use of A1c is now appropriately referred to as HbA1c.

6. **In the expression “p varied from...,” if the authors are referring to a confidence interval, this should be reported as: p < 0.0001; 95% CI: 0.0001–0.003.**

The reviewer's question is very important and extremely pertinent regarding this unusual form of analysis.

One of the constant criticisms made when comparing continuous or categorical data in large samples is that significant differences occur between samples with minimum mean/median values that have no clinical relevance. The same occurs with the comparison between rates that may present significant differences between minimum percentage differences that also have no clinical relevance.

To eliminate this statistical bias, in the comparisons mentioned by the reviewer, an important resource was used: the analysis of random subsamples within the samples studied. In this case, the standard of one thousand subsamples was used, which, because they have a smaller number of individuals, mitigates this bias and also excludes the possibility of potential subsamples with a different behavior from the sample as a whole. Therefore, for each comparison of these subsamples, a significance value (p) is generated, resulting in a thousand significance values. To determine whether any of these sub-analyses have a discrepancy, the confidence intervals of the thousand significances analyzed are calculated. For example: if a lower confidence interval value were found with a significance of less than 0.05 while the upper confidence interval value was greater than 0.05, we would have a null statistical analysis.

An alternate method for determination of the accuracy of the model parameters is the so-called “bootstrap” method (Efron, 1979; Efron and Tibshirani, 1993; Shao and Tu, 1996).

Andrych et al. (2020) and Andronov and Kulynska (2020) discussed statistical properties of the approximations of the “bootstrap-generated” data sets in more detail.

**References (bootstrapping algorithms)**

•	Sheen Mclean Cabaneros, Ben Hughes, Methods used for handling and quantifying model uncertainty of artificial neural network models for air pollution forecasting,Environmental Modelling & Software,Volume 158,2022,105529,ISSN 1364-8152, (https://doi.org/10.1016/j.envsoft.2022.105529)

•	Davison, A. C., and D. V. Hinkley. 2006. Bootstrap Methods and their Application. : Cambridge University Press.

•	Shao, J., and D. Tu. 1995. The Jackknife and Bootstrap. New York: Springer.

7. **Lines 256–258: The statement regarding the “misperception of high Lp(a) as a rare genetic disorder” is unclear and potentially misleading. Why should elevated Lp(a) be perceived as a rare genetic disorder? However, I do not believe this is the main reason for the limited assessment of Lp(a) in clinical practice. Rather, it likely reflects restricted access to laboratory testing, limited awareness among clinicians, and—perhaps most importantly—the current lack of approved therapies.**

Thank you for your thought-provoking comments. We appreciate your constructive perspective.

Our objective with the statement regarding the "misperception of high Lp(a) as a rare genetic disorder" was to highlight the common misconception that elevated Lp(a) levels are confined to a small group of individuals with genetic disorders. This overlooks the fact that elevated Lp(a) levels are more widespread, affecting nearly 1 in 5 individuals when levels surpass 50 mg/dL.

That being said, you are absolutely right that the limited assessment of Lp(a) in clinical practice may also be due to factors such as restricted laboratory testing, limited awareness of Lp(a) among clinicians, and the lack of approved therapies specifically targeting Lp(a).

In light of your feedback, we will refine our thesis. We aim to impart that misconceptions about the rarity of Lp(a) dyslipidemia, as well as the factors you outlined, significantly contribute to the underutilization of Lp(a) assessments in routine clinical practice.

8. **Tables: Please use a dot (.) instead of a comma (,) for decimal separation.**

Thank you for pointing that out. We have standardized the decimal format throughout the tables, replacing commas with dots, as per your suggestion.

9. **Units of measurement are missing in Table 1 and should be added.**

Thank you for your observation. Units of measurement were added to the table caption.

10. **There are two tables labeled as Table 2—please correct the numbering.**

Thank you for your comment. We reviewed the table numbers to ensure clarity and consistency.

11. **Please check and revise the legend of Table 3 for clarity and consistency.**

Thank you for your observation. The table was redesigned to improve clarity and consistency.
